# Ownership of dogs and cats leads to higher levels of well-being and general trust through family involvement in late adolescence

**DOI:** 10.3389/fvets.2023.1220265

**Published:** 2023-08-31

**Authors:** Hikari Koyasu, Sakura Ogasawara, Takefumi Kikusui, Miho Nagasawa

**Affiliations:** Laboratory of Human-Animal Interaction and Reciprocity, Department of Animal Science and Biotechnology, Azabu University, Sagamihara, Kanagawa, Japan

**Keywords:** dog, cat, adolescent, family, well-being, general trust

## Abstract

**Introduction:**

Late adolescence is a crucial period during which individuals connect with new communities. Furthermore, their mental health has lasting effects on their overall well-being. Involvement with family and the local community plays a significant role in shaping adolescents’ personalities and well-being. Additionally, pets, such as dogs and cats, function as social catalysts and increase interactions with family and the local community. We hypothesized that pet ownership would increase involvement with family and the local community and thereby impact adolescents’ personalities and well-being.

**Methods:**

Therefore, this study investigated whether owning dogs or cats was related to well-being through increased involvement with family and local community members in late adolescence. Data were collected via a questionnaire administered to high school and university students. The questionnaire included questions on basic information about adolescents and their families, pet ownership experience, family and local community involvement, well-being, cultural estrangement inventory, and general trust.

**Results:**

Structural equation modeling revealed that adolescent women who owned dogs or cats had higher well-being and general trust through their involvement with their families. Although previous research reported that men who had experienced pet ownership in childhood were more sociable in old age, the effect of pet ownership on men was not observed in this study.

**Discussion:**

During late adolescence, when individuals experience many connections with new communities, the effects of pets may temporarily decrease. Therefore, future cohort studies should examine the effects of pets on each age group.

## 1. Introduction

During the transition from late adolescence to early adulthood, individuals often encounter several risks and opportunities (1). Health and mental issues that arise during this period can have a lasting impact on an individual’s overall well-being (2). Patton et al. (3) found that mental health disorders during adolescence were the strongest predictors of the same in young adulthood. Additionally, the values and beliefs during adolescence influenced their well-being in older age (4). These findings suggested that an individual’s personality and health status had long-term effects on their overall well-being. Japan’s mental health ranks 37th out of the 38 developed countries (5). The percentage of adolescents who feel comfortable making friends is low at 68%, which ranks also 37th (5). Low mental health among adolescents is a serious concern in Japan. Elucidating the factors that influence their well-being and personality could help address this issue.

During late adolescence, individuals form relationships extending beyond familial connections and thus develop their personalities. Environmental factors such as social relationships are relatively stable constituents that influence personality (6). In Japan, a notable personality characteristic is the low level of general trust (7). General trust is a personality of belief in the benevolence of human nature in general and thus is not confined to specific relations. A study has shown that the Japanese are more likely to distrust others than Americans and have fewer opportunities to establish new relationships (7). Conflicts arising from personality and value differences are not exclusive to family members but also occur among friends and others. Individuals who perceive themselves as misaligned with community values may experience self-discrepancy, potentially leading to life dissatisfaction, depression, low self-esteem, and interpersonal anxiety (8).

As adolescents expand their social connections beyond the family unit, the social environment that influences well-being shifts. Maintaining strong ties with family members, particularly parents, is crucial for adolescent well-being (9). Studies have consistently shown, across diverse cultures, that adolescents who are highly engaged with their families experience greater life satisfaction and reduced psychological distress (10). As these individuals progress into late adolescence and spend more time interacting with friends and non-family members, the influence of these relationships on their well-being becomes increasingly evident. Adolescents who maintain higher levels of involvement with friends and neighbors also demonstrate high well-being (11). Thus, during adolescence, interactions with family members, friends, and neighbors are pivotal factors contributing to an individual’s well-being.

Pets function as social catalysts that facilitate people-to-people relationships. Walsh (12) suggests that pets can serve as a significant factor in unifying families and mitigating family conflicts. Studies have demonstrated that the presence of a pet enhances interpersonal interactions (13, 14). Beyond the context of familial relationships, pets also contribute to the development of social bonds within local communities. It has been observed that pet owners are more likely to be acquainted with their neighbors compared to non-pet owners, with 40% of pet owners reporting receiving social support from relationships made through their pets (15, 16). Specifically, dog owners were found to be five times more likely to establish new social connections compared to owners of other types of pets (15). In addition, children who had recently acquired a pet dog were reported to have visited more friends during a one-month follow-up compared to children without a dog (17). Through dog walking, dogs can facilitate interactions and help individuals form new relationships (18, 19). People with dogs are often perceived as amiable (20), and dogs serve as effective icebreakers in social situations (15, 21–23). Owing to the necessity of outdoor activities, such as walking, dogs exert a broad impact on interpersonal relationships. In contrast, there are few reports on the social relationships of cat owners. Notably, cat owners who demonstrate a high level of attachment to their pets report lower levels of social support, although the causal direction of this relationship remains unclear (24). Pets, particularly dogs, can potentially facilitate the establishment of connections with others, thereby contributing to enhanced well-being.

Recently, extensive research has been conducted on the relationship between pet ownership (e.g., dogs and cats) and well-being. Many studies have reported that pets are beneficial to people, including adolescents (25). Studies have also reported that owning pets has a positive impact on well-being, regardless of species (26–29). However, other findings suggest that pet species, such as dogs and cats, have different effects on well-being (15, 28, 30, 31). A cohort study in Japan found that adolescents who owned dogs had a consistently high level of well-being from the ages of 10–12 years (31). Conversely, adolescents who owned cats experienced a significant decrease in well-being compared to those who owned dogs or had no pets (31).

**Figure 1:**
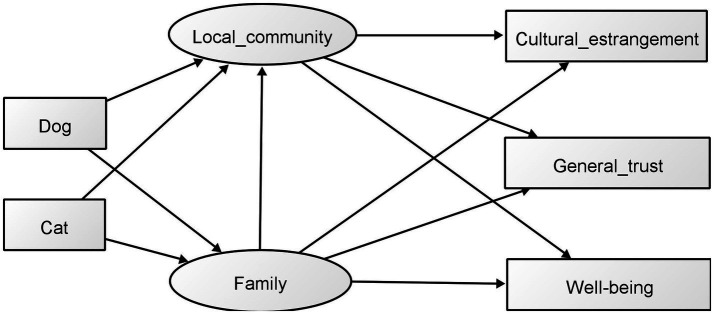
Hypothetical model. “Dog” and “Cat” indicate the experience of owning dogs and cats.

Based on these findings, we hypothesized that the ownership of a dog or cat forms a late adolescent’s personality, such as general trust, and boosts well-being in late adolescence through the involvement of family and local communities (Figure 1). This study aimed to examine the above-stated hypotheses via structural equation modeling (SEM) using questionnaire survey.

## 2. Materials and methods

The survey included questions regarding the participants’ basic attributes, family composition, pet ownership, involvements with family members and the local community, as well as indicators of cultural estrangement inventory, general trust, and well-being measured using the World Health Organization-Five Well-Being Index. Questions regarding animal attitudes and attachment to pets were also included; however, these were not analyzed.

### 2.1. Subjects

An online survey was conducted via Cross Marketing Inc. (Tokyo, Japan). Data screening was conducted by the survey company. This screening process targeted only high school and university students and excluded careless responses. We used 2,845 data points for the analysis.

### 2.2. Participants’ basic attributes

The participants answered questions regarding their sex, age, prefecture of residence, occupation (high school or university student), type of residence (apartment or house), and annual household income.

### 2.3. Family composition

The survey included questions regarding the participants’ family composition, such as the age of family members and participants’ parents’ jobs.

### 2.4. Pet ownership

The participants’ past and current pet ownership experiences were also recorded. This included the type of pet owned (dog, cat, or other), where the pet was kept, and the amount of time they spent interacting with the pet when they were in elementary, middle, and high school, as well as university.

### 2.5. Cultural estrangement inventory

To assess the degree of alignment between one’s own values and those of one’s family and surroundings, the Japanese version of the Cultural Estrangement Inventory (CEI) developed by Cozzarelli and Karafa (8) was used. The CEI was primarily designed to measure cultural estrangement tendencies in the American culture and consisted of five items each related to culturally atypical and misfit constructs. In this study, the questions were adapted to replace “Japanese people” or “people in this country” with “family,” “friends/neighbors,” and “community members” (e.g., “I strongly identify with my family’s values”). All questions were answered on a 7-point Likert scale ranging from 1 (strongly disagree) to 7 (strongly agree). The Cronbach’s alpha coefficient, indicating the reliability between items, was 0.904 for atypical and 0.883 for misfit, and the total of the atypical and misfit constructs was 0.821. In this study, the total of atypical and misfit was used for the present study’s analysis.

### 2.6. World Health Organization-five well-being index

The World Health Organization developed the Five Well-Being Index (WHO-5) as a simple indicator of mental health. The Japanese version of the WHO-5 was developed by Awata et al. (32, 33) after its equivalence with the original version was confirmed and its procedures were standardized. The WHO-5 consists of five questions that enquire about an individual’s moods, such as “during the past two weeks, how often have you been in good spirits?” It has the advantage of being able to measure mental health status within a short period. Responses were rated on a 5-point Likert scale ranging from 1 (all of the time) to 5 (none of the time). The scores were summed across the five questions. The raw scores ranged from 0 to 25 points, with 0 and 25 indicating the poorest and best well-being status, respectively. The Cronbach’s alpha coefficient was 0.910.

### 2.7. General trust

General trust was measured using items developed by Yamagishi (7, 34, 35). The participants were asked to rate their agreement with statements, such as “most people can be trusted,” “people who are trusted tend to trust others,” “most people are trustworthy,” “most people are fundamentally honest,” “I tend to trust people,” and “most people are basically kind and helpful” on a 7-point Likert scale that ranged from 1 (strongly disagree) to 7 (strongly agree). The Cronbach’s alpha coefficient was 0.905.

### 2.8. Involvement with family

The participants answered three questions regarding their relationships with their families: the amount of time they spent talking with their family (F1), the frequency of conversations regarding themselves with their family (F2), and the amount of time they spent together in a family gathering place (F3) such as a living room. The responses were rated on a 4-point scale. The Cronbach’s alpha coefficient was 0.807.

### 2.9. Involvement with local community

The participants answered questions regarding their frequency of community activities in the neighborhood (LC1); activities that involved interaction with others, such as sports, hobbies, and lessons (LC2); volunteering (LC3); and meeting with classmates outside of school (LC4). Regarding the frequency of meeting with classmates, they answered on a 5-point Likert scale ranging from 1 (never) to 5 (daily). For the other questions, the responses were rated on a 7-point Likert scale ranging from 1 (not participating) to 7 (more than four times a week). The Cronbach’s alpha coefficient was 0.572.

### 2.10. Statistical analysis

Pet ownership was coded as follows: owning a dog was coded as 1, not owning a dog was coded as 0; owning a cat was coded as 1, not owning a cat was coded as 0. This coding was applied for both current and past pet ownership. These were treated as four separate variables. T-tests were performed to evaluate the presence of differences between individuals in well-being, general trust, and CEI across several categories, including: gender (male vs. female), sibling status (presence vs. absence of siblings), type of residence (house vs. apartment), current or past dog ownership (groups with vs. without dogs), and current or past cat ownership (groups with vs. without cats). If adolescents owned both dogs and cats, due to the potential that they were influenced by owning both types of pets, the data were overlapped in the analysis. Pearson’s correlation coefficients were used to investigate the relationship between well-being, general trust, and CEI and age, income, and involvement with family and the local community. The significance level was set at 5%. These analyses were performed using JMP^®^ (ver. 14.2.0). Subsequently, we performed SEM using the WLSMV method with the lavaan package in R (ver. 4.2.1). The relatively low Cronbach’s alpha coefficients for family and community involvement were speculated to be because they comprised only three and four items, respectively. Consequently, for SEM, we used the individual item values, rather than the aggregate scores, for both family and community involvement. Sex was an important risk factor for mental health disorders. Females were diagnosed with depression and anxiety disorders at much higher rates than males in early adolescence (36) and had lower well-being (37, 38). Therefore, we conducted separate analyses based on sex because different developmental processes could result in different processes related to pet ownership, family relationships, community involvement, and well-being. To control for socioeconomic status, additional analysis was conducted with the model including income. As more than 50% of the respondents did not know their family income and the fit index of the model was low [comparative fit index (CFI): 0.823–0.859, Tucker–Lewis index (TLI): 0.754–0.803, root mean square error of approximation (RMSEA): 0.058–0.067, and standardized root mean squared residual (SRMR): 0.063–0.074], the model was adopted without income. For the observed variables of family relationships and community involvement, we added covariance relationships with modification indices greater than five and adopted the final model to improve statistical validity without losing logical validity.

## 3. Results

### 3.1. Descriptive statistics

Among the respondents, 753 were men and 2092 were women. There were 1,033 high school students and 1812 university students. Furthermore, 2,110 people had siblings. Regarding residence type, 1,625 and 1,220 people lived in houses and apartments, respectively. Regarding pet ownership, 773, 592, and 1,480 people currently owned pets, previously owned pets, and never owned pets, respectively. Of those who currently owned pets, 396 owned dogs, 230 owned cats, and 230 owned other pet types. In addition, 415 participants owned dogs, 245 owned cats, and 634 owned other types of pets (some reported ownership of multiple pets). Other descriptive statistics are shown in the Supplementary material.

### 3.2. Correlation analysis

#### 3.2.1. Well-being

Significant sex differences were observed, with males scoring higher than females [*t* (2843) = −2.285, *p* = 0.024]. Current dog ownership was negatively associated with well-being, and those who currently owned dogs scored lower than those who did not [*t* (2843) = −2.280, *p* = 0.023]. Similarly, individuals who had previously owned a dog scored lower than those who had never owned one [*t* (2843) = −2.052, *p* = 0.040]. Additionally, annual income, local community involvement, and family involvement were significantly correlated with well-being. Higher income was positively associated with well-being (rs = 0.132, *p* < 0.001), as were greater levels of community involvement (community: rs = 0.108, *p* < 0.001, rs = 0.155, *p* < 0.001, rs = 0.119, *p* < 0.001, rs = 0.211, *p* < 0.001, rs = 0.226, *p* < 0.001) and family involvement (family: rs = 0.162, *p* < 0.001, rs = 0.193, *p* < 0.001, rs = 0.140, *p* < 0.001, rs = 0.196, *p* < 0.001). These results are included in the Supplementary material.

#### 3.2.2. General trust

We examined the effects of having siblings, current dog ownership, and previous dog ownership. Those with siblings scored higher than those without them [*t* (2843) = 2.617, *p* = 0.009]. Furthermore, those who currently owned dogs had lower scores than those who did not [*t* (2843) = −2.629, *p* = 0.009], as did those who had owned dogs in the past compared to those who had never owned them [*t* (2843) = −2.190, *p* = 0.028]. Furthermore, there was a positive correlation between general trust and age (*r* = 0.062, *p* < 0.001). There were significant positive correlations among general trust, community involvement, and family involvement (community, LC1: rs = 0.038, *p* = 0.040; LC2: rs = 0.120, *p* < 0.001; LC3: rs = 0.057, *p* = 0.002; LC4: rs = 0.161, *p* < 0.001; family, F1: rs = 0.081, *p* < 0.001; F2: rs = 0.130, *p* < 0.001; F3: rs = 0.073, *p* = 0.004). These results are included in Supplementary material.

#### 3.2.3. Cultural estrangement inventory

The CEI score was higher for males than for females [*t* (2843) = −3.352, *p* = 0.022], and for high school students than for university students [*t* (2843) = −2.295, *p* < 0.001]. In addition, significant negative correlations were found with age (rs = −0.061, *p* = 0.001) and income (rs = −0.084, *p* = 0.004). Negative correlations were also found with family (talk time: rs = −0.071, *p* < 0.001; talk about oneself: rs = −0.126, *p* < 0.001; sharing space: rs = −0.065, *p* < 0.001) and community involvement (hobby: rs = −0.078, *p* < 0.001; classmate: rs = −0.164, *p* < 0.001). These results are included in the Supplementary material.

#### 3.2.4. Family and local community involvement

Regarding family involvement, sex, residence type, current dog or cat ownership, age, and income were significantly related. Women were more involved with their families than men [*t* (2843) = 10.395, *p* < 0.001]. Furthermore, those living in houses had higher involvement than those living in apartments [*t* (2843) = −9.494, *p* < 0.001]. People who owned dogs and cats had a higher level of involvement than those who did not [*t* (2843) = 2.379, *p* = 0.017; *t* (2843) = 2.532, *p* = 0.011]. Furthermore, a negative correlation was observed between age and involvement (rs = −0.138, *p* < 0.001), while, a positive correlation was observed between income and involvement (rs = 0.218, *p* < 0.001).

Local community involvement was significantly related with sex, presence of siblings, past dog ownership, age, and income. Community involvement was higher among men than among women [*t* (2843) = −1.999, *p* = 0.046], and among those with siblings compared to those without [*t* (2843) = 2.130, *p* = 0.033]. Those who previously owned dogs were also more likely to have a relationship with the local community [*t* (2843) = 2.915, *p* = 0.004]. In addition, there was a negative correlation between age and relationship with the local community (rs = −0.117, *p* < 0.001), while, there was a positive correlation between income and the relationship with the local community (rs = 0.096, *p* < 0.001).

A positive correlation was observed between family and community involvement (rs = 0.146, *p* < 0.001).

Considering the connections between these factors, it was possible to explain the hypothesis that owning a dog or cat led to greater involvement with the family and community, which resulted in increased well-being, general trust, and CEI. However, when the direct relationship between owning dogs or cats and well-being, general trust, and CEI was examined, a negative effect was observed for dog ownership, whereas no effect was observed for cat ownership. Additionally, the correlation coefficients were low for items that showed correlations. Well-being, general trust, and CEI could not be explained by a single factor, as each factor was complexly intertwined and generated; this possibility was also considered in the correlation analysis. Therefore, SEM was undertaken based on the hypothesis model.

### 3.3. SEM

The effects of the current and past ownership of dogs and cats were also examined. All models showed a good fit with the following indices: CFI > 0.95, TLI > 0.92, RMSEA<0.05, and SRMR <0.04. The goodness of fit for each model is shown in Table 1.

**Table 1:** Fitness of each model.

Model	Chi2	CFI	TLI	RMSEA	SRMR
Dogcat_now_male	76.134	0.968	0.950	0.033	0.032
Dogcat_now_female	174.917	0.958	0.934	0.039	0.029
Dogcat_before_male	52.751	0.990	0.984	0.019	0.025
Dogcat_before_female	175.839	0.957	0.933	0.039	0.030

#### 3.3.1. Effects of current ownership of dogs and cats

**Figure 2:**
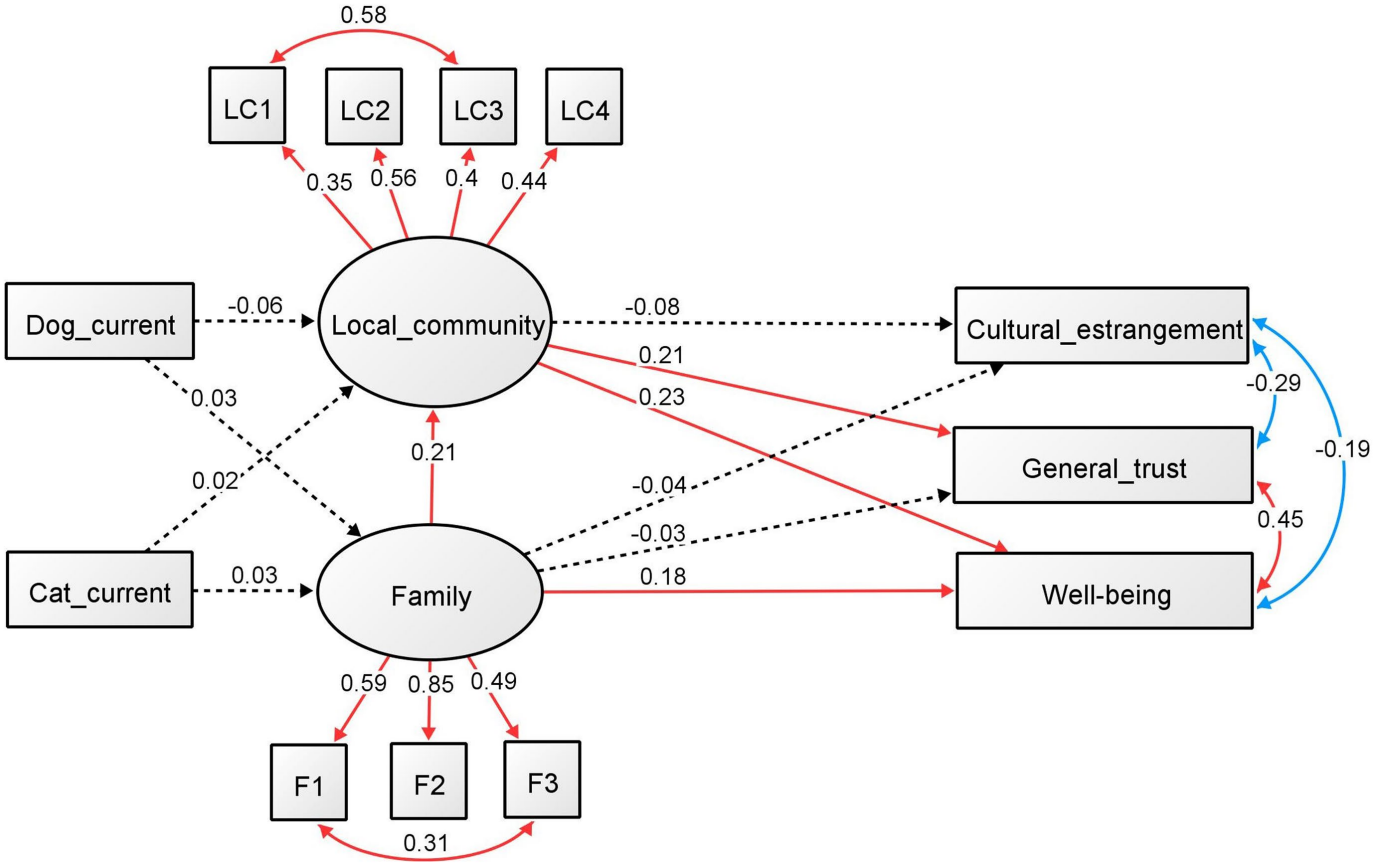
SEM of the effects of the current ownership of dogs and cats (males). Standardized regression coefficients and factor loadings between the components are shown.

The results for males and females are shown in Figures 2 3, respectively. The standardized regression coefficients and factor loadings between the components are also shown in each figure. For men (Figure 2), the effect of current dog and cat ownership on the family and local community was not significant. The regression analyses that were significant were from family involvement to local community involvement (standardized *ß* = 0.205, *p* = 0.002) and well-being (standardized *ß* = 0.179, *p* = 0.001), and from local community involvement to well-being (standardized *ß* = 0.233, *p* < 0.001) and general trust (standardized *ß* = 0.208, *p* = 0.002). The other regression analysis was not significant. All factors were significant in the results of the factor analysis for family and community involvement (*p* < 0.001). The standardized factor loadings for family involvements were 0.595 for talk time (F1), 0.847 for talk about oneself (F2), and 0.496 for sharing space (F3), whereas for local community involvement, the values were 0.350 for community activity (LC1), 0.564 for hobby (LC2), 0.400 for volunteer (LC3), and 0.443 for classmate (LC4). In addition, all covariance relationships, as added with the modified index, were significant (*p* < 0.001). The standardized estimates were 0.577 for community activity (LC1) and volunteer (LC3), −0.112 for volunteer (LC3) and classmate (LC4), 0.308 for family talk time (F1) and space sharing (F3), −0.289 for CEI and general trust, −0.187 for CEI and well-being, and 0.453 for general trust and well-being.

**Figure 3:**
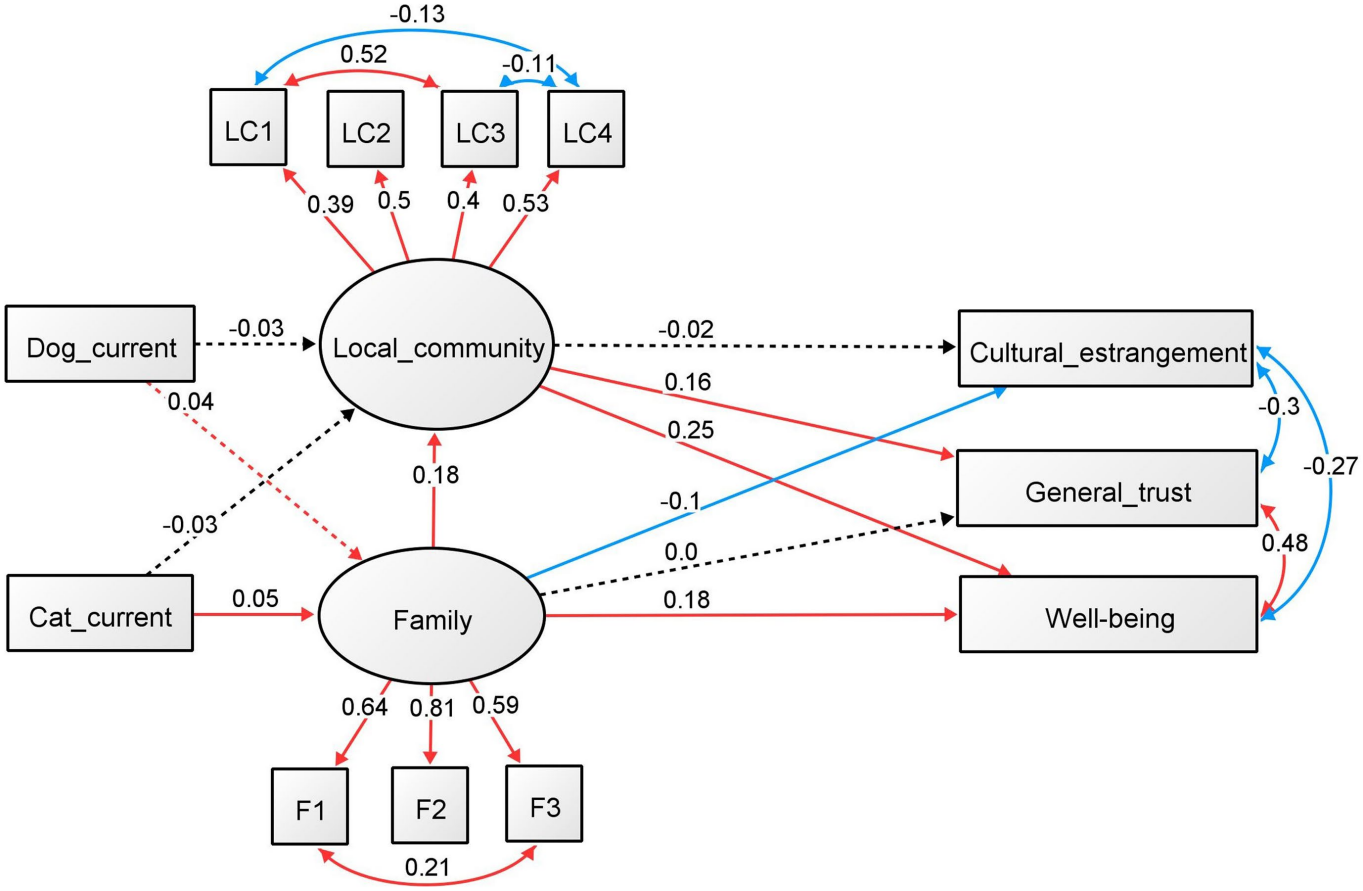
SEM on the effects of current ownership of dogs and cats (females). Standardized regression coefficients and factor loadings between the components are shown.

For women (Figure 3), current dog and cat ownership predicted family involvement (dog: standardized *ß* = 0.043, *p* = 0.078; cat: standardized *ß* = 0.053, *p* = 0.032). The relationship between dog and cat ownership and local community involvement was not significant. The regression analyses that were significant were from family involvement to local community involvement (standardized *ß* = 0.178, *p* < 0.001), CEI (standardized *ß* = −0.101, *p* < 0.001), and well-being (standardized *ß* = 0.184, *p* < 0.001). Involvement with the local community was also significantly related to well-being (standardized *ß* = 0.248, *p* < 0.001) and general trust (standardized *ß* = 0.159, *p* < 0.001). The other regression analysis was not significant. All factors were significant in the results of the factor analysis for family and community involvement (*p* < 0.001). The standardized factor loadings for family involvements were 0.641 for talk time (F1), 0.809 for talk about oneself (F2), and 0.586 for sharing space (F3), while for local community involvement, the values were 0.391 for community activity (LC1), 0.504 for hobby (LC2), 0.403 for volunteer (LC3), and 0.532 for classmate (LC4). In addition, all covariance relationships, as added with the modified index, were significant (*p* < 0.001). The standardized estimates were 0.577 for community activity (LC1) and volunteer (LC3), 0.308 for family talk time (F1) and space sharing (F3), −0.289 for CEI and general trust, −0.187 for CEI and well-being, and 0.453 for general trust and well-being. Moreover, all the covariance relationships, as added with the modified index, were significant (*p* < 0.001). The standardized estimates were 0.520 for community activity (LC1) and volunteer (LC3), −0.131 for community activity (LC1) and classmate (LC4), 0.210 for family talk time (F1) and space sharing (F3), −0.304 for CEI and general trust, −0.267 for CEI and well-being, and 0.483 for general trust and well-being.

#### 3.3.2. Effects of past ownership of dogs and cats

**Figure 4:**
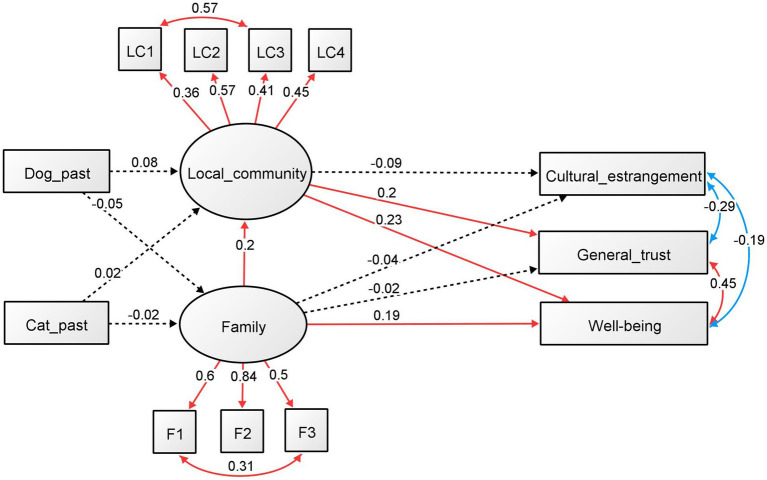
SEM on the effects of past ownership of dogs and cats (males). Standardized regression coefficients and factor loadings between the components are shown.

The results for males and females are shown in Figures 4 5, respectively. The standardized regression coefficients and factor loadings between the components are also shown in each figure.

**Figure 5:**
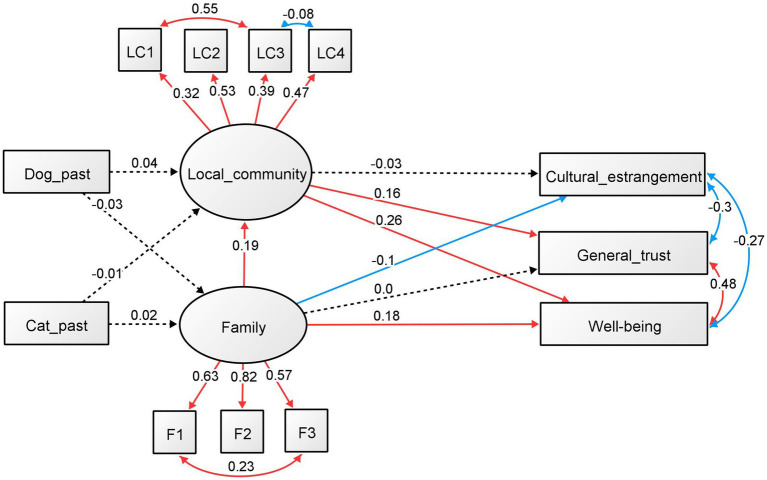
The standardized regression coefficients and factor loadings between the components are shown.

For men (Figure 4), the effect of past dog and cat ownership on the family and local community was not significant. Family involvement was significantly associated with local community involvement (standardized *ß* = 0.199, *p* = 0.003) and well-being (standardized *ß* = 0.186, *p* = 0.001). Local community involvement was significantly associated with well-being (standardized *ß* = 0.228, *p* < 0.001) and general trust (standardized *ß* = 0.199, *p* = 0.002). The other regression analysis was not significant. All factors were significant in the results of the factor analysis for family and community involvement (*p* < 0.001). The standardized factor loadings for family involvements were 0.600 for talk time (F1), 0.840 for talk about oneself (F2), and 0.497 for sharing space (F3), while for local community involvement, the values were 0.355 for community activity (LC1), 0.570 for hobby (LC2), 0.405 for volunteer (LC3), and 0.445 for classmate (LC4). In addition, all the covariance relationships, as added with the modified index, were significant (*p* < 0.001). The standardized estimates were 0.573 for community activity (LC1) and volunteer (LC3), 0.305 for family talk time (F1) and space sharing (F3), −0.289 for CEI and general trust, −0.187 for CEI and well-being, and 0.453 for general trust and well-being.

For women (Figure 5), the effect of past dog and cat ownership on the family and local community was not significant. Family involvement was significantly associated with local community involvement (standardized *ß* = 0.187, *p* < 0.001), CEI (standardized *ß* = −0.104, *p* < 0.001), and well-being (standardized *ß* = 0.183, *p* < 0.001). The other regression analysis was not significant. All factors were significant in the results of the factor analysis for family and community involvement (*p* < 0.001). The standardized factor loadings for family involvements were 0.628 for talk time (F1), 0.821 for talk about oneself (F2), and 0.573 for sharing space (F3), while for local community involvement, the values were 0.324 for community activity (LC1), 0.532 for hobby (LC2), 0.391 for volunteer (LC3), and 0.471 for classmate (LC4). In addition, all covariance relationships, as added with the modified index, were significant (*p* < 0.001). The standardized estimates were 0.553 for community activity (LC1) and volunteer (LC3), −0.078 for volunteer (LC3) and classmate (LC4), 0.226 for family talk time (F1) and space sharing (F3), −0.304 for CEI and general trust, −0.267 for CEI and well-being, and 0.483 for general trust and well-being.

Details on these effects are included in Table 2.

**Table 2:** Summary of the effects of structural equation modeling.

	Current ownership						Past ownership					
	Male			Female			Male			Female		
	Standardized estimate	SE	*p*	Standardized estimate	SE	*p*	Standardized estimate	SE	*p*	Standardized estimate	SE	*p*
**Regressions**												
Dog→Family	0.025	0.045	0.577	**0.043**	**0.024**	**0.078**	−0.053	0.047	0.259	-0.027	0.025	0.279
Dog→Local community	−0.062	0.047	0.191	−0.035	0.028	0.263	0.084	0.051	0.103	0.040	0.031	0.195
Cat→Family	0.033	0.046	0.473	**0.053**	**0.025**	**0.032**	−0.022	0.048	0.649	0.019	0.026	0.451
Cat→Local community	0.017	0.054	0.747	−0.032	0.028	0.263	0.023	0.050	0.640	−0.015	0.030	0.620
Family→CEI	−0.045	0.046	0.323	−**0.101**	**0.029**	**0.078**	−0.044	0.046	0.336	−**0.104**	**0.029**	**0.000**
Family→Well-being	**0.179**	**0.054**	**0.001**	**0.184**	**0.032**	**0.000**	**0.186**	**0.054**	**0.001**	**0.183**	**0.032**	**0.000**
Family→General trust	−0.031	0.056	0.578	0.002	0.032	0.938	−0.023	0.056	0.677	0.001	0.032	0.967
Family→Local community	**0.205**	**0.068**	**0.002**	**0.178**	**0.037**	**0.000**	**0.199**	**0.067**	**0.003**	**0.187**	**0.039**	**0.000**
Local community→CEI	−0.088	0.059	0.136	−0.025	0.032	0.435	−0.089	0.058	0.125	−0.027	0.034	0.438
Local community→Well-being	**0.233**	**0.064**	**0.000**	**0.248**	**0.037**	**0.000**	**0.228**	**0.063**	**0.000**	**0.255**	**0.039**	**0.000**
Local community→General trust	**0.208**	**0.066**	**0.002**	**0.159**	**0.037**	**0.000**	**0.199**	**0.066**	**0.002**	**0.164**	**0.039**	**0.000**
**Latent variables**												
Family→F1	**0.595**	**0.062**	**0.000**	**0.641**	**0.029**	**0.000**	**0.600**	**0.061**	**0.000**	**0.600**	**0.061**	**0.000**
Family→F2	**0.847**	**0.091**	**0.000**	**0.809**	**0.036**	**0.000**	**0.840**	**0.089**	**0.000**	**0.840**	**0.089**	**0.000**
Family→F3	**0.496**	**0.059**	**0.000**	**0.586**	**0.029**	**0.000**	**0.497**	**0.058**	**0.000**	**0.497**	**0.058**	**0.000**
Local community→LC1	**0.350**	**0.048**	**0.000**	**0.391**	**0.044**	**0.000**	**0.355**	**0.049**	**0.000**	**0.324**	**0.034**	**0.000**
Local community→LC2	**0.564**	**0.046**	**0.000**	**0.504**	**0.031**	**0.000**	**0.570**	**0.045**	**0.000**	**0.532**	**0.030**	**0.000**
Local community→LC3	**0.400**	**0.049**	**0.000**	**0.403**	**0.044**	**0.000**	**0.405**	**0.050**	**0.000**	**0.391**	**0.039**	**0.000**
Local community→LC4	**0.443**	**0.049**	**0.000**	**0.532**	**0.042**	**0.000**	**0.445**	**0.049**	**0.000**	**0.471**	**0.036**	**0.000**
**Covariances**												
LC1↔LC3	**0.577**	**0.068**	**0.000**	**0.520**	**0.041**	**0.000**	**0.573**	**0.067**	**0.000**	**0.573**	**0.067**	**0.000**
LC1↔LC4				−**0.131**	**0.034**	**0.000**						
LC3↔LC4				−**0.112**	**0.036**	**0.002**				−**0.078**	**0.026**	**0.002**
F1↔F3	**0.308**	**0.068**	**0.000**	**0.210**	**0.037**	**0.000**	**0.305**	**0.068**	**0.000**	**0.305**	**0.068**	**0.000**
CEI↔General trust	−**0.289**	**0.044**	**0.000**	−**0.304**	**0.026**	**0.000**	−**0.289**	**0.044**	**0.000**	−**0.289**	**0.044**	**0.000**
CEI↔Well-being	−**0.187**	**0.038**	**0.000**	−**0.267**	**0.024**	**0.000**	−**0.187**	**0.038**	**0.000**	−**0.187**	**0.038**	**0.000**
Well-being↔General trust	**0.453**	**0.044**	**0.000**	**0.483**	**0.026**	**0.000**	**0.453**	**0.044**	**0.000**	**0.453**	**0.044**	**0.000**

Results that were statistically significant are in bold.

## 4. Discussion

We hypothesized that owning dogs or cats during late adolescence would have high involvements with family and community, which relate with individual personality and well-being. To test this hypothesis, we conducted a questionnaire survey and SEM.

According to the SEM results, among females, those who currently owned dogs or cats in late adolescence had more family involvement. Family involvement also affected their local community involvement positively. However, no relationship was observed between owning dogs or cats and local community involvement. Adolescents owning dogs or cats have high family involvement. In addition, adolescents that had previously owned dogs had high levels of local community involvement. However, in the SEM, a significant effect was observed only for current ownership of dogs and cats. The absence of any observed effect of past ownership on the SEM could be explained as follows. When multiple factors were considered, the impact of other factors could outweigh that of owning dogs or cats, leading to relatively small effects.

Including certain factors, such as conversation time and content, as well as time spent in the living room with family and increased time spent together in the same room with pets as a central focus, may have led to increased connections with the family by owning dogs or cats. Previous research has reported that pet ownership in families of children with autism spectrum disorder improves family functioning (39). This study suggests that owning pets in the families of adolescent females with no or minimal emotional or developmental difficulties may improve family functioning through increased connections between adolescents and their families.

The effects of owning dogs and cats through family and community involvement on well-being was different from in the Tokyo Teen Cohort Study on cat ownership (31). While the Tokyo Teen Cohort Study found negative effects of cat ownership on adolescent children (31), this study also found positive effects of owning cats. One possible explanation for this difference is age, as suggested by Poresky et al. (40). According to their study, the self-concept scores of individuals aged 14–49 years were influenced by the age at which they first owned a pet (40). Individuals who owned pets before the age of 5 years or during adolescence (12–15 years old) had higher positive, physical, and social scores on the Tennessee Self-Concept Scale than those who started owning pets between 6 and 11 years of age (40). Additionally, it has been posited that emotional support from cats can act as a substitute for social support from people (24). Several studies have also demonstrated that pet ownership can alleviate feelings of loneliness and social isolation (27, 41–43). These findings imply that pet ownership may diminish the need to interact extensively with others by reducing feelings of loneliness. Furthermore, the depth of attachment to pets differs depending on the family structure (44, 45), and adolescents’ attachment to pets affects their family relationships (29). Therefore, it is crucial to consider variables such as timing of pet ownership, depth of attachment to pets, and degree of interaction with pets to enhance our understanding of their impact.

Interestingly, the factors that influenced the CEI differed between males and females. For males, local community involvement increased their degree of value alignment with their surroundings through ties with the local community. In contrast, in females, both family and local community involvement increased their degree of value alignment with their surroundings. Communities could play a significant role in shaping personality development among males and females in different ways. Previous studies reported that social networks differed between males and females, and females had more family centered networks, while males had more non-kin networks centered on colleagues (46). Even in adolescence, boys have larger friend networks than girls (47). Furthermore, during childhood, boys met their friends more frequently and invested more in them than girls did (48). Women who emphasized close-knit communities had a greater influence on the formation of their values.

Both men and women demonstrated positive effects of local community involvement on general trust. People with low levels of general trust tended to limit their opportunities to engage in new communities by confining their interactions to small networks (34). The transition from late adolescence to adulthood is a period during which people engage in new communities. However, during this period, individuals with limited interactions with their friends and the community, and those who remained in small networks were more likely to have low levels of general trust.

Both men and women showed positive effects on well-being through their involvement with their families and communities. Having meals with family members was linked to high levels of adolescent well-being (49–54). Family, community, and friends involvement enhanced well-being. The strength of social cohesion in the local community was linked to a lower likelihood of experiencing depression and anxiety symptoms in adolescents (55), suggesting that creating a socially cohesive local community could lead to increased well-being. Additionally, friendships and social networks could provide social support for dealing with problems encountered in social life and alleviate perceived stress (56, 57).

This study has several limitations. First, it is a cross-sectional study. Therefore, the causal effects of pet ownership remain unclear. Second, there was bias in the participants’ demographic characteristics. The participants were not randomly selected. They were recruited from among individuals who had registered with a survey company, which might have biased the results toward individuals with a higher interest in surveys. Third, the proportion of women was higher than that of men, and male data accounted for less than half of the female data. The analysis was conducted separately for men and women, which might have led to Type II errors. Therefore, future studies should use larger sample sizes to reduce demographic bias and improve statistical power. Although health status was an important factor involved in well-being, it was excluded from this survey. Given our teenage target demographics, we anticipated that a substantial number of respondents would not report significant health issues. Furthermore, to avoid overwhelming the participants, we limited the number of questionnaire items. However, this decision certainly warrants further reflection. Additionally, the survey did not analyze the impacts of pet species other than dogs and cats. This could be attributed to the fact that we did not inquire about the specific species of pets owned by the participants. A comparison between the effects of dog and cat ownership and the ownership of various other species could provide valuable insights into the unique characteristics of these popular pet types.

Notwithstanding these limitations, this study revealed that late adolescent women who owned a dog or cat had high involvement with their family, which resulted in higher well-being. However, no significant effects were observed among men. Previous studies reported that men’s sociability increased with pet ownership and greater interactions during childhood (58). It was possible that the effect of pets temporarily declined among men with broader networks, particularly during late adolescence when the experiences of social connections were more diverse. Further cohort studies are required to track the effects of pets on different age groups.

## Data availability statement

The original contributions presented in the study are included in the article/Supplementary material, further inquiries can be directed to the corresponding author.

## Ethics statement

The studies involving humans were approved by Ethical Committee for Medical and Health Research Involving Human Subjects of Azabu University. The studies were conducted in accordance with the local legislation and institutional requirements. Written informed consent for participation in this study was provided by the participants’ legal guardians/next of kin.

## Author contributions

HK, SO, TK, and MN contributed to the conception and design of the study. HK, SO, and MN organized the database. HK and SO performed the statistical analysis. HK, TK, and MN acquired funding and wrote the first draft of the manuscript and revised it accordingly. All authors contributed to the article and approved the submitted version.

## Funding

This research was supported by the Japan Society for the Promotion of Science and Grants-in-Aid for Scientific Research from the Ministry of Education, Culture, Sports, Science, and Technology of Japan; 21H05173 (MN) and 23H05472 (TK), supported by the JST-Mirai Program; JPMJMI21J3 (TK) and the Center for Diversity, Equity and Inclusion, Azabu University (HK).

## Conflict of interest

The authors declare that the research was conducted in the absence of any commercial or financial relationships that could be construed as a potential conflict of interest.

## Publisher’s note

All claims expressed in this article are solely those of the authors and do not necessarily represent those of their affiliated organizations, or those of the publisher, the editors and the reviewers. Any product that may be evaluated in this article, or claim that may be made by its manufacturer, is not guaranteed or endorsed by the publisher.
